# Psychosocial risk factors for headache in medical students

**DOI:** 10.1192/j.eurpsy.2024.1352

**Published:** 2024-08-27

**Authors:** E. L. Nikolaev, F. V. Orlov, S. S. Fakhraei

**Affiliations:** ^1^Department of Social and Clinical Psychology; ^2^Department of Psychiatry, Medical Pscyhology and Neurology; ^3^Medical Faculty, Ulianov Chuvash State University, Cheboksary, Russian Federation

## Abstract

**Introduction:**

Headache is often considered as a symptom reflecting mental ill-being of a person. Taking into account heavy academic loads, we should study it in medical students in reference to its connections with various psychosocial risk factors

**Objectives:**

To establish interrelations between the frequency of headaches in medical students and risk factors of psychosocial nature

**Methods:**

We conducted the research based on the Faculty of Medicine of Ulianov Chuvash State University. It covered 546 students of both genders who had no complains of having mental problems. We surveyed the students by means of Sociocultural Health Questionnaire (E. Nikolaev)

**Results:**

The research showed that two out of three students complained of headaches of various intensity and frequency. It was present with statistically equal frequency (p>.05) in domestic (68.85%) and foreign (63.90%) medical students. Females experience headache more often (r=.20), and it more often correlates with a high level of stress (r=.25), lesser satisfaction with studying (r=-.14), higher frequency of e-cigarette consumption (r=.15), higher anxiety due to phantom ringing syndrome (r=.15), lower self-esteem of health (r=-.29), confidence (r=-.16), successfulness (r=-.12), happiness (r=-.18), well-being (r=-.11), liveliness (r=-.16), higher frequency of medication consumption (r=.27), higher frequency of visits to a psychotherapist in the childhood (r=.11), higher current need in the help of a psychologist (r=.21), psychiatrist and psychotherapist (r=.21).

**Conclusions:**

These psychosocial risk factors call for attention from mental health professionals, and we should take them into consideration while providing medical care to medical students and developing health programs in universities.

**Disclosure of Interest:**

None Declared

